# Spatial Difference of Interactive Effect Between Temperature and Daylength on Ginkgo Budburst

**DOI:** 10.3389/fpls.2022.887226

**Published:** 2022-05-10

**Authors:** Zhaofei Wu, Shuxin Wang, Yongshuo H. Fu, Yufeng Gong, Chen-Feng Lin, Yun-Peng Zhao, Janet S. Prevéy, Constantin Zohner

**Affiliations:** ^1^College of Water Sciences, Beijing Normal University, Beijing, China; ^2^Systematic & Evolutionary Botany and Biodiversity Group, MOE Key Laboratory of Biosystems Homeostasis & Protection, College of Life Sciences, Zhejiang University, Hangzhou, China; ^3^WSL Institute for Snow and Avalanche Research SLF, Davos, Switzerland; ^4^Institute of Integrative Biology, ETH Zurich (Swiss Federal Institute of Technology), Zurich, Switzerland

**Keywords:** climate change, daylength, spatial variation, latitude, twig-cutting experiment, gymnosperms

## Abstract

Climate warming-induced shifts in spring phenology have substantially affected the structure and function of terrestrial ecosystems and global biogeochemical cycles. Spring phenology is primarily triggered by spring temperature and is also affected by daylength and winter chilling, yet the relative importance of these cues across spatial gradients remains poorly understood. Here, we conducted a manipulative experiment with two daylength and three temperature treatments to investigate spatial differences in the response of ginkgo budburst to temperature and daylength, using twigs collected at three sites across a spatial gradient: a control site at a low latitude and low elevation on Tianmu Mountain (TM_low_), a low latitude and high elevation site on Tianmu Mountain (TM_high_), and a high latitude site on Jiufeng mountain (JF). The mechanisms were also tested using *in situ* phenological observations of ginkgo along latitudes in China. We found that, compared to TM_low_ individuals, budburst dates occurred 12.6 (JF) and 7.7 (TM_high_) days earlier in high-latitude and high-elevation individuals when exposed to the same temperature and daylength treatments. Importantly, daylength only affected budburst at low latitudes, with long days (16 h) advancing budburst in low-latitude individuals by, on average, 8.1 days relative to short-day (8 h) conditions. This advance was most pronounced in low-elevation/latitude individuals (TM_low_ = 9.6 days; TM_high_ = 6.7 days; JF = 1.6 days). In addition, we found that the temperature sensitivity of budburst decreased from 3.4 to 2.4 days °C^−1^ along latitude and from 3.4 to 2.5 days °C^−1^ along elevation, respectively. The field phenological observations verified the experimental results. Our findings provide empirical evidence of spatial differences in the relative effects of spring temperature and daylength on ginkgo budburst, which improved our understanding of spatial difference in phenological changes and the responses of terrestrial ecosystem to climate change.

## Introduction

The ongoing shifts in vegetation phenology resulting from climate change substantially affect carbon, water, and energy fluxes (Buermann et al., 2018; Piao et al., 2019a; Zhou et al., 2020; Wu et al., 2022a). Spring phenology, in particular, has attracted widespread attention as it marks the onset of the growing season and photosynthesis (Korner and Basler, 2010; Fu et al., 2015; Zohner et al., 2016; Piao et al., 2019b; Zhang et al., 2021). It has been reported that the length of vegetation growing season is a primary contributor for the carbon uptake (Piao et al., 2017) and the net carbon uptake increases by 4.5 kg ha^−1^ for per 1 day earlier of the spring phenology (Keenan et al., 2014). Therefore, understanding how environmental triggers regulate spring phenology of plants is critical to improve our ability to forecast the effects of climate change on terrestrial ecosystems (Keenan et al., 2014; Piao et al., 2019b). It has been widely reported that, as a result of warmer spring conditions, climate change has led to significant advances in spring phenology over recent decades (Piao et al., 2019b; Menzel et al., 2020; Zhuqiu et al., 2021). As plants continue to leaf out earlier, daylength may become an increasingly important factor, limiting warming-induced advances in spring phenology (Basler and Körner, 2012; Way and Montgomery, 2015; Fu et al., 2019a). However, how daylength and temperature interact to trigger spring phenology, and how these interactive signals differ across spatial gradients, remain largely unknown (Zohner et al., 2016; Piao et al., 2019a; Wu et al., 2022b).

Studies that focus on the interactive effects of daylength and temperature (Fu et al., 2019b) often find that plants require more cumulative heat (forcing requirement) until budburst when days are still short (Korner and Basler, 2010; Basler and Körner, 2012; Way and Montgomery, 2015). This response can be seen as a safety mechanism to minimize the risk of frost damage that would arise from precocious budburst. The daylength effect is species-specific (Basler and Körner, 2012; Zohner and Renner, 2015), and previous experimental studies found a wide range of responses from being insensitive to daylength to showing no budburst at all under short days (Zohner and Renner, 2015; Zohner et al., 2016). These studies mostly focused on inter-specific comparisons within angiosperms, while population-level studies that also involve gymnosperms are scarce (but see Kumar and Sati, 2016; Pan et al., 2021; Wu et al., 2022b).

Phenological timing and responsiveness to the underlying environmental drivers evolved as an adaptation to local climates (Peaucelle et al., 2019). Responses of spring phenology to climate change can thus be expected to differ substantially across spatial gradients as a result of population- and species-level differences in the environmental stimuli governing phenological timing for both deciduous broad-leaved (Lechowicz, 1984; Peaucelle et al., 2019; Zhuqiu et al., 2020) and evergreen conifer species (Hänninen, 1995; Salmela et al., 2011, 2013; Ma et al., 2018). For example, Wu et al. (2022b) found that the sensitivity of spring phenology to the climate drivers such as temperature and daylength decreases with elevation. However, similar temperature sensitivity was found along elevation among geographically separated populations of European tree species (Vitasse et al., 2009). How the response of budburst to temperature and daylength along elevational gradients thus remains under debate? In addition, previous study found that species from lower latitudes appear to rely on daylength and temperature as budburst signals, while species from high-latitudes flush independent of daylength, instead relying on the length of winter and spring warming as signals (Zohner et al., 2016). However, experimental studies on spatial, especially latitudinal, variations in the importance of temperature and daylength on spring budburst within species are scarce, but integral to improving our ability to forecast phenological timing across space and time.

Ginkgo (*Ginkgo biloba* L.), the so-called living fossil, is an early diverged lineage of gymnosperms and is widely distributed across temperate areas in East Asia (Major, 1967; Zhou and Zheng, 2003; Peter, 2007). Here, we conduct a manipulative twig-cutting experiment to investigate spatial variations in the responsiveness of ginkgo budburst to temperature and daylength. Twig cuttings have been shown to provide realistic proxies of the phenological responses of adult trees to changes in temperature and daylength (Laube et al., 2013; Menzel et al., 2020; Zohner et al., 2021). We collected twigs from three sites: a low-latitude and low-elevation site, a low-latitude and high-elevation site, and a high-latitude and low-elevation site. In addition, the natural datasets derived from Chinese Phenological Observation Network (CPON) were also used to verify the results of the twig-cutting experiment. We aim to test the following three hypotheses that (1) both temperature and daylength affect the budburst of ginkgo; (2) budburst occurs earlier in twigs from high latitudes and elevations under common temperature and daylength conditions due to a smaller heat requirement; and (3) the temperature sensitivity of budburst would be affected by daylength, and its effect is spatially different.

## Materials and Methods

### *In situ* Phenological Observation

**Figure 1:**
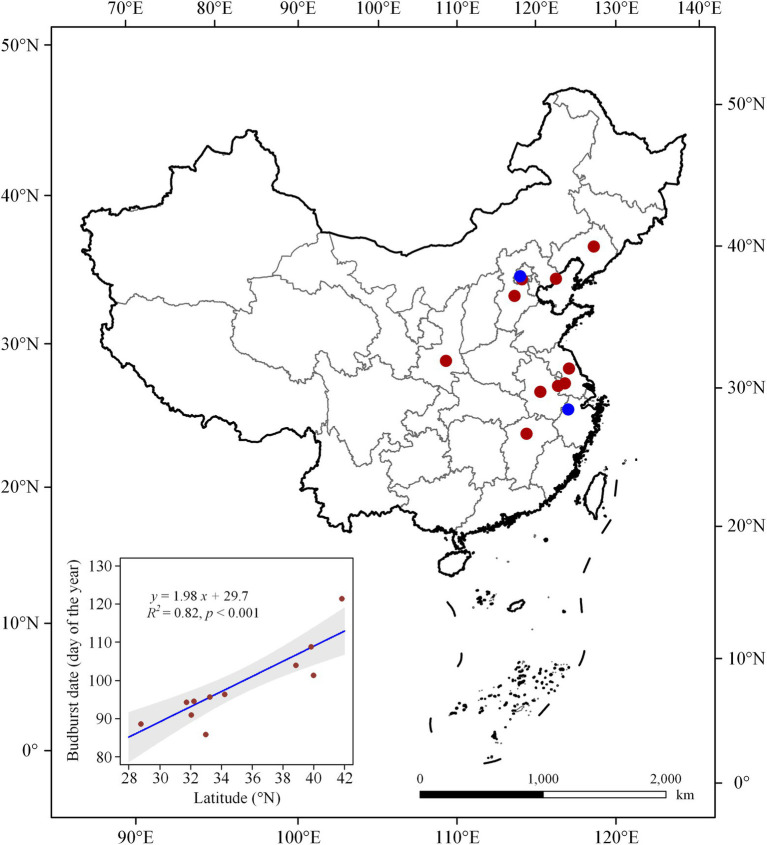
The geospatial distribution of the *in situ* observational sites (red dots) and the collection sites of the twigs (blue dots). The inner figure shows the latitudinal variation in budburst dates.

Chinese Phenological Observation Network (CPON)^1^ was developed in 1963, which was widely used in phenological studies (Ge et al., 2015). In the present study, we selected sites where ginkgo has been observed for more than 10 years. In total, 10 sites in east China were selected and the spring budburst dates were used in our analyzation (Figure 1; Supplementary Table S1). The climate data were derived from China Meteorological Forcing Dataset^2^, which was developed by Data Assimilation and Modeling Center for Tibetan Multi spheres, Institute of Tibetan Plateau Research, Chinese Academy of Sciences (He et al., 2020). In the current study, we defined the preseason as 2 months prior to the mean date of budburst for each site following previous study (Fu et al., 2016) and further calculated the mean temperature during the preseason.

### Twig Collections

To investigate spatial variation in the effect of temperature and daylength on gingko budburst, we conducted twig-cutting experiments in climate chambers. We collected twigs of ginkgo from three sites: a high-latitude site at Jiufeng Mountain (JF), Beijing in North China (366 m; 116° 28′ E, 39° 54′ N), and two low-latitude sites differing in elevation at Tianmu Mountain (TM), Zhejiang Province in East China (119° 26′ E, 30°19′ N, high-elevation site, TM_high_ = 1,105 m; low-elevation site, TM_low_ = 347 m). TM is characterized by subtropical evergreen and deciduous broad-leaved mixed forest, while JF is characterized by temperate broad-leaved deciduous forest.

Twigs were collected from adult ginkgo trees on January 17–22, 2021 at the above three sites. In total, 29 individual trees were selected, of which 11 and 12 individuals came from TM_low_ and TM_high_, respectively, and 6 individuals from JF (this was the maximum possible number due to the management policy at the site; see details in Table 1). Six twigs, approximately 40 cm in length, were cut from each individual. The twigs were cleaned and disinfected with commercial hypochlorite solution following previous studies (Du et al., 2019; Wu et al., 2022b) and then cultivated in 395 ml plastic bottles filled with tap water. Every 2 weeks from the start of the experiment, the tap water was changed in bottles, the twigs were washed to remove mold grown, and their basal parts were trimmed by about 2 cm to avoid vessel occlusion.

**Table 1:** Details of the selected individual trees.

Sites			Latitude	Elevation	Number of the selected individuals	Breast height diameter (cm)
Tianmu Mountain	Low-latitude	TM_low_	30°19′ N	347	12	28.1 ± 20.0
	Low elevation					
Tianmu Mountain	Low-latitude	TM_high_	30°19′ N	1,105	11	32.4 ± 18.8
	High elevation					
Jiufeng Mountain	High latitude	JF	39° 54’ N	366	6	24.7 ± 3.3

### Manipulative Experiment in Climate Chambers

Three climate chambers were used to manipulate air temperature, and daylength treatments were set up in each temperature treatment by covering half of the twigs with shade black cloth. Following a 2 × 3 full-factorial design, two daylength treatments (8-h [P8] and 16-h daylength [P16]) were combined with three temperature treatments (10°C [T10], 15°C [T15], and 20°C [T20]). To avoid plant variations within populations, the twigs of each specific individual were separately put into six environmental treatments (3 temperature × 2 daylength). In total, 174 twigs were used in this experiment, of which, per treatment, 6 twigs (replicates) came from JF, 12 twigs from TM_low_, and 11 twigs from TM_high_.

Temperature sensors (HOBO M2202) were installed within each environmental treatment. No significant difference in temperature was found between the two daylength treatments by using the ANCOVA analysis (Supplementary Figure S1). Following Vitasse (2013), the budburst date was defined as the date when buds start to open and leaves become partially visible. The twigs were put into the chambers on January 23, 2021. We monitored buds of each twig every 3 days during the treatment period. The days to achieve budburst (BBD) were defined as the number of days from treatment start (January 23, 2021) until budburst.

### Data Analysis

For the *in situ* phenological observations, changes in budburst date and the mean preseason temperature along the latitudinal gradient were estimated by using linear regression analysis. In addition, we conducted a correlation analysis to investigate the relationship between budburst date and preseason temperature at each site, and further explored the latitudinal variation of the correlation coefficients between budburst date and preseason temperature. As a stronger daylength effects also associated with a lower variation in budburst dates, we thus estimated the daylength limitation effect by using the standard deviation of budburst dates (Std) as a surrogate measure (Zohner et al., 2016; Geng et al., 2022). For the twig-cutting experiment, the heat requirement for budburst was calculated as the cumulative growing degree days (GDD) from January 23, when the twigs were put into the chambers, until budburst date:


$$GDD=\overset{\mathrm{Budburst}}{\underset{\mathrm{Jan}.23}{\sum }}\left(T_{day}-T_{base}\right)\;if\;T_{day}>T_{base}$$

where *T*_day_ is the mean daily temperature and *T*_base_ is the base temperature. Following previous studies, 0°C was used as *T*_base_ (Sarvas, 1972; Fu et al., 2016).

Differences of BBD and GDD between the temperature and daylength treatments were tested using independent samples t-tests. We also calculated chilling days (CHD) when the daily temperature was between −10 and 7°C from 1 September 2020 to the starting date of the experiment (Weinberger, 1950; Wang et al., 2020). The temperature sensitivity of budburst (*S*_T_) was defined as the days advance of budburst date per degree warming (days °C^−1^), which was calculated using linear regression analysis (Fu et al., 2015). Differences in temperature sensitivity between the daylength treatments were tested using ANCOVA (Fu et al., 2019a). All statistical analyses were conducted using R version 3.5.2.

## Results

### Latitudinal Variation of Spring Budburst

Based on the *in situ* observations, we found that the spatial distribution of budburst dates followed a strong latitudinal pattern (Figure 1). Budburst dates (day of year, DOY) varied from DOY 89 at the southernmost Nanchang to DOY 122 at the northernmost Shenyang. For every 1° increase in latitude, DOY delayed by 1.98 days (Figure 1).

### Spatial Differences in Temperature Responses of Budburst

**Figure 2:**
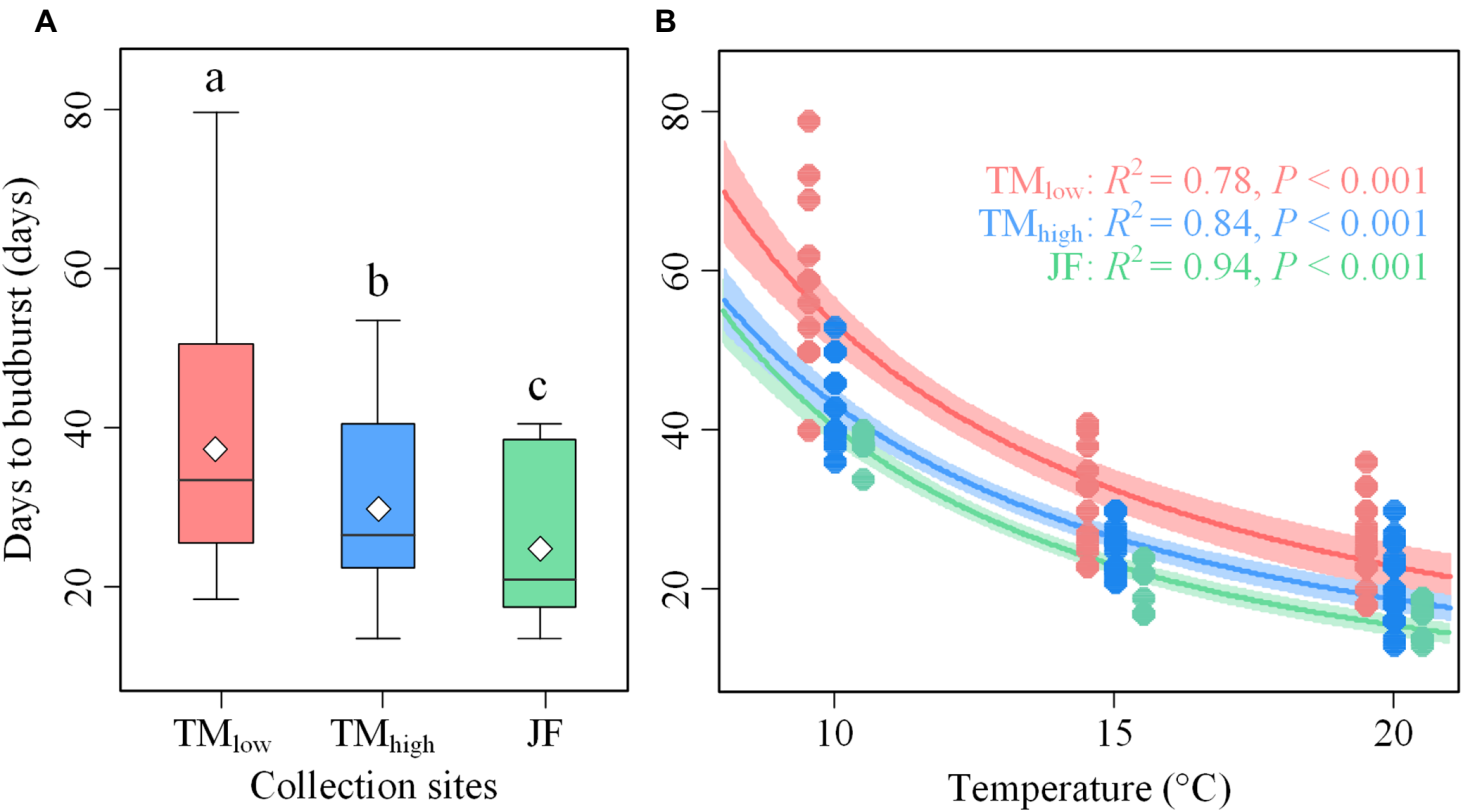
**(A)** Differences in the days to budburst (BBD) under controlled conditions between the collection sites. **(B)** The relationship between BBD and treatment temperature for twigs from the three collection sites. Different letters in **(A)** denote significant differences (*p* < 0.05) in BBD between the collection sites. TM_low_, TM_high_, and JF refer to the low-elevation and high-elevation collection site in Tianmu Mountain (low latitude) and the collection site in Jiufeng Mountain (high latitude), respectively.

In our experiment, 83.3% (145) of the 174 twigs achieved budburst, and these were used for subsequent analysis. Under the same temperature and daylength regime, twigs collected from the high-latitude site (JF) were the first ones to flush, while twigs from the low-latitude/low-elevation site (TM_low_) were the last ones to flush, with low-latitude/high-elevation twigs intermediate (TM_high_, Figure 2A). In detail, the twigs collected at JF and TM_high_ showed, on average, 12.6 and 4.9 days earlier budburst, respectively, than those from TM_low_.

Budburst became significantly earlier with the increase in temperature across treatments (Figure 2B). Compared with the T10 temperature treatment, budburst occurred 20.6 and 25.9 days (*p* < 0.05) earlier in the T15 and T20 treatments, respectively. The largest effect of temperature treatment was found for TM_low_ twigs (32.1 days difference between T10 and T20), followed by TM_high_ (23.5 days) and JF twigs (22.1 days), respectively.

### Spatial Difference in Responses of Budburst to Daylength

**Figure 3:**
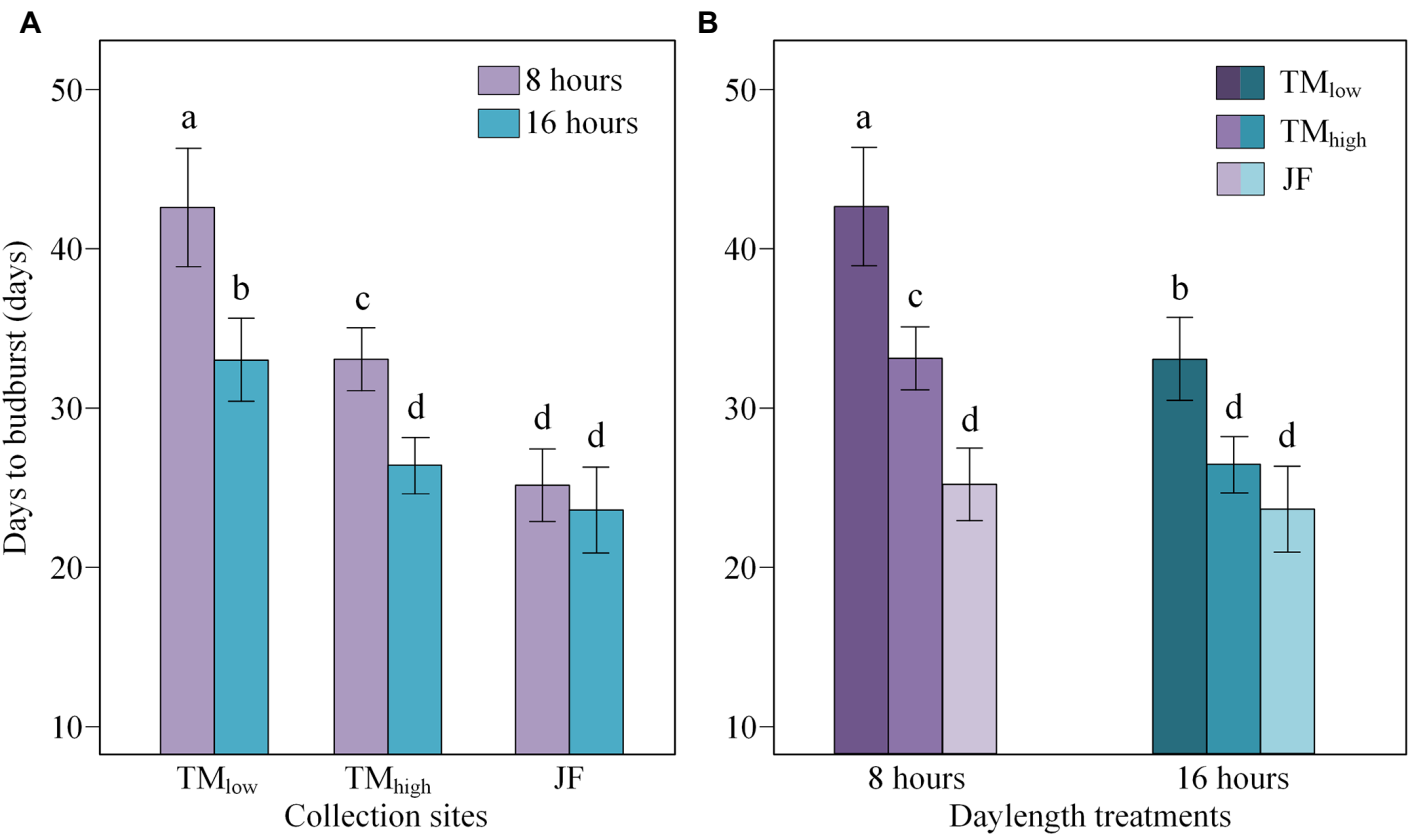
**(A)** Effects of daylength on the days to budburst for twigs from the three collection sites. **(B)** Same as panel **(A)** but grouped by daylength treatment. A 8 and 16 h refer to the daylength treatments in the climate chambers. TM_low_, TM_high_, and JF refer to the low-elevation and high-elevation collection site in Tianmu Mountain (low latitude) and the collection site in Jiufeng Mountain (high latitude), respectively. Different letters denote significant difference (*p* < 0.05) in BBD between the combinations of collection site and daylength treatment.

Long daylength significantly advanced budburst in twigs collected at the two low-latitude sites, i.e., relative to 8-h short-day conditions, budburst occurred 9.6 and 6.7 days earlier under 16-h long days in TM_low_ and TM_high_ twigs, respectively (*p* < 0.05; Figure 3A). However, daylength did not affect budburst dates in twigs from the high-latitude site (JF), i.e., BBD = 23.6 and 25.1 days for the 16-h and 8-h treatments (*p* = 0.66). Under both 8-h and 16-h daylength, budburst occurred earliest in JF twigs, latest in TM_low_ twigs, with TM_high_ twigs intermediate (Figure 3B). Long-day conditions largely reduced the phenological differences among twigs from the three sites relative to 8-h short-day conditions (Figure 3B).

### Interactive Effect of Temperature and Daylength on Budburst

**Figure 4:**
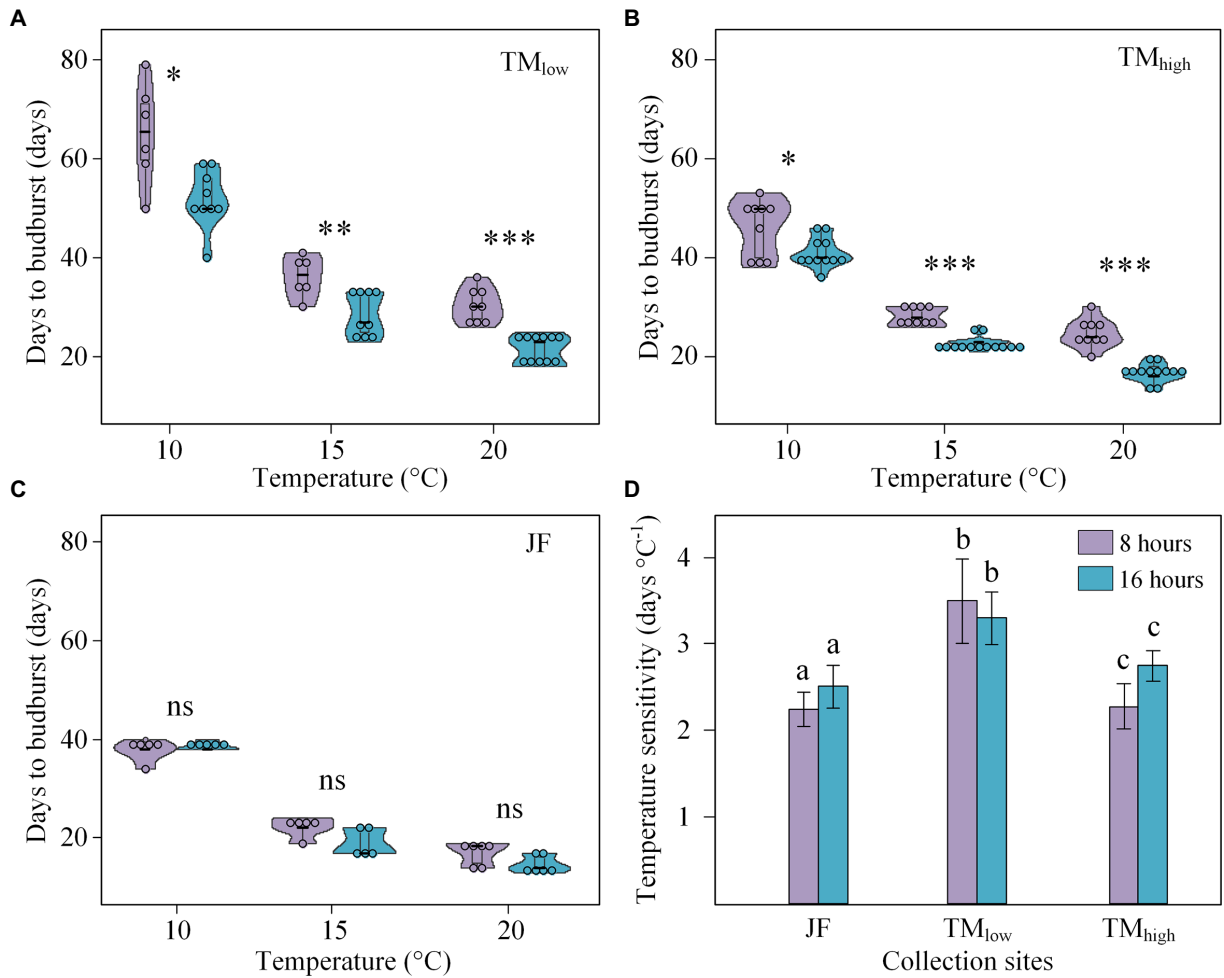
Interactive effects of temperature and daylength on the days to budburst (BBD) for TM_low_ **(A)**, TM_high_ **(B)**, JF **(C)**, and the temperature sensitivity (*S*_T_) of budburst **(D)**. TM_low_, TM_high_, and JF refer to the low-elevation and high-elevation collection site in Tianmu Mountain (low latitude) and the collection site in Jiufeng Mountain (high latitude), respectively. A 8 and 16 h refer to the daylength treatments in the climate chambers. ^***^, ^**^, and ^*^ indicate significant differences between the daylength treatments and collection sites at 0.001, 0.01, and 0.05 level. *ns* referred to no significant was found. Different letters in **(D)** denote significant differences (*p* < 0.05) in *S*_T_ of the collection sites and the daylength treatments.

In agreement with the above results, increased temperature consistently advanced budburst, while longer daylength only advanced budburst in twigs from the two low-latitude sites for all three temperature treatments (Figures 4A–C). Interestingly, the daylength effect was greater under warmer conditions with a higher significance level in the T20 treatment (*p* < 0.001), compared to the T10 treatment (*p* < 0.05) at both TM_high_ and TM_low_ (Figures 4A,B). We further found that temperature sensitivity of budburst significantly decreased with latitude and elevation, ranging from 3.98 days °C^−1^ (TM_low_) to 2.53 days °C^−1^ (TM_high_) and 2.38 days °C^−1^ (JF). However, no significant difference in the temperature sensitivity of budburst was found when compared between the two daylength treatments (Figure 4D).

**Figure 5:**
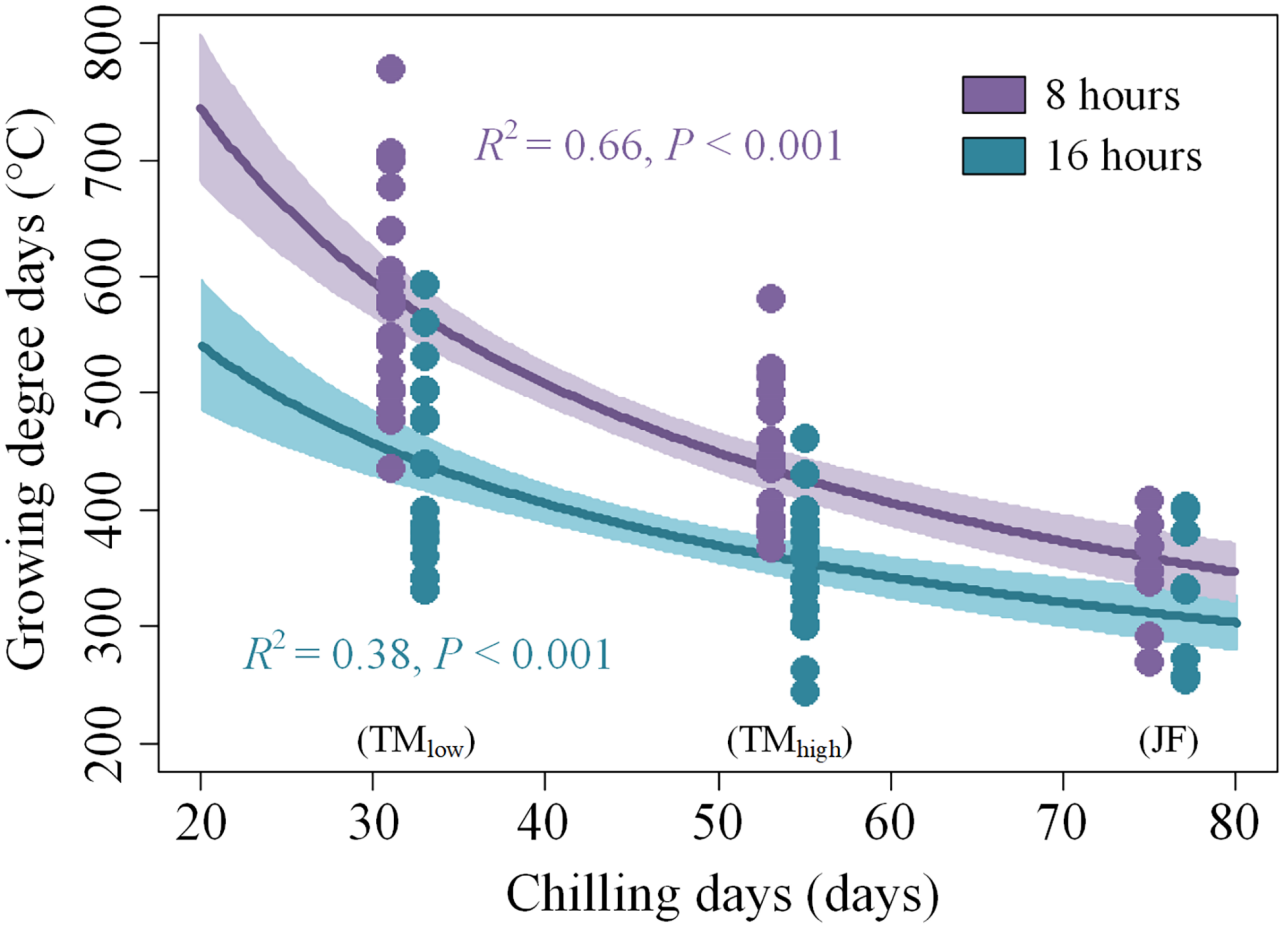
The relationship between growing degree days and chilling days. A 8 and 16 h refer to the daylength treatments in the climate chambers. TM_low_, TM_high_, and JF refer to the low-elevation and high-elevation collection site in Tianmu Mountain (low latitude) and the collection site in Jiufeng Mountain (high latitude), respectively.

As shown in Figure 5; Supplementary Figure S2, the chilling days increased from low elevation/latitude to high elevation/latitude (JF > TM_high_ > TM_low_). Chilling significantly reduced the growing degree days (GDD) required for budburst under both daylength treatments (Figure 5). Among the three sites, twigs from JF had the lowest GDD requirement (336°C) to release budburst; the highest GDD requirement was found for the low-latitude/low-elevation site (TM_low_: 500°C), with the low-latitude/high-elevation site being intermediate (TM_high_: 389°C, Figure 5). Daylength significantly reduced the forcing requirement at the two low-latitude sites, yet no significant daylength effect was found at JF (Figure 5; Supplementary Figure S2). Long daylength reduced the GDD requirement by 24.0 and 21.6% for TM_low_ and TM_high_, respectively (Figure 5).

**Figure 6:**
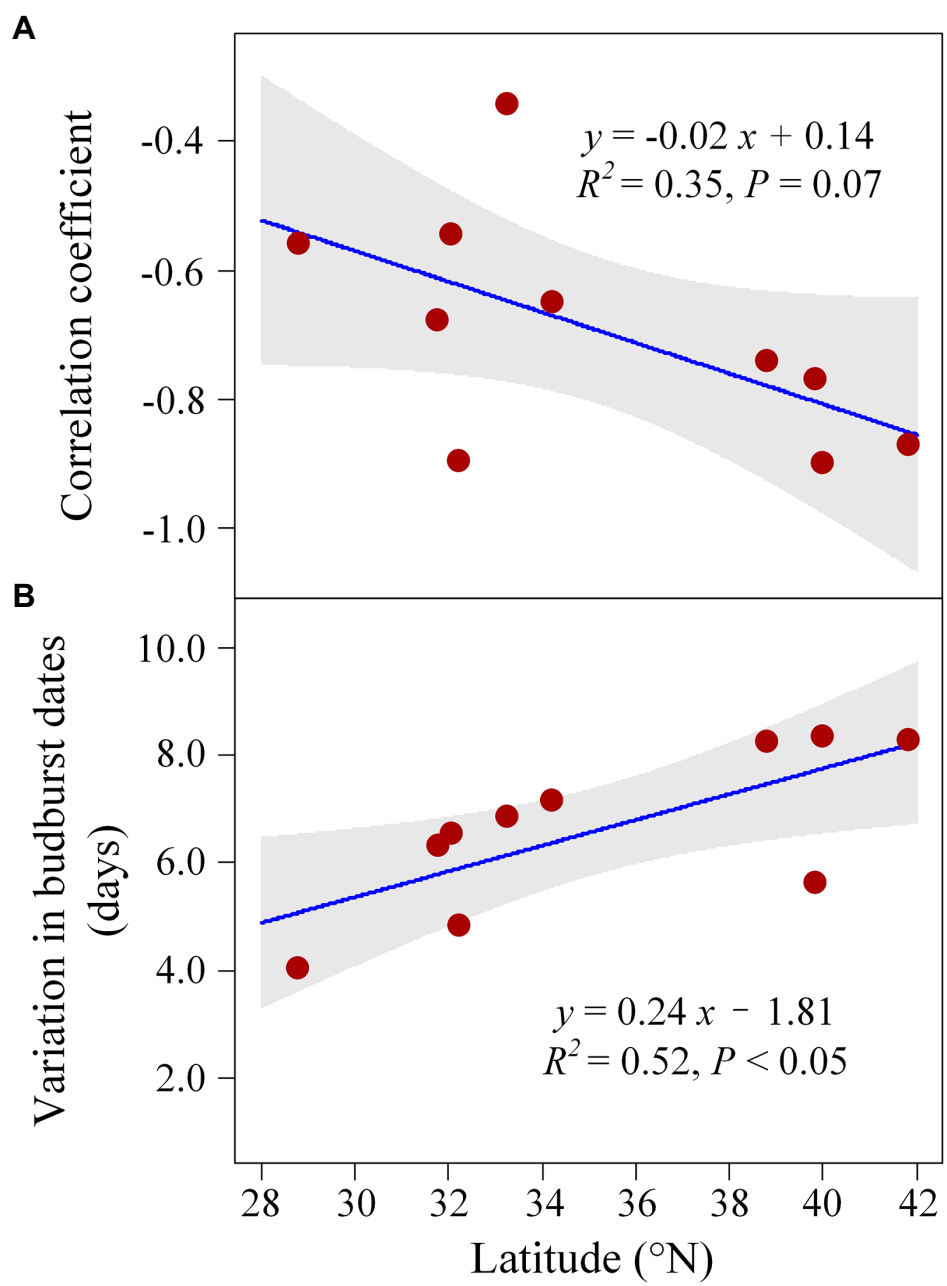
Shifts of the correlation coefficient between preseason temperature and budburst **(A)** and the variation in budburst dates **(B)** along latitudes.

Consistent with the above results, the *in situ* phenological observations showed that spring budburst and temperature are negatively correlated across all sites. The negative relationship (correlation coefficient) between spring budburst and temperature strengthens along latitudes at a rate of −0.02 °N^−1^ (Figure 6A), suggesting a high temperature controls on ginkgo budburst at high latitudes.

## Discussion

### Latitudinal Patterns of Spring Budburst of Ginkgo

In accordance with Hopkins’ bioclimatic law, the budburst timing of ginkgo was delayed with increasing latitude (Hopkins, 1920; Wang et al., 2015; Meng et al., 2021), driven by decreases in preseason temperature (Supplementary Figure S3). However, while Hopkins proposed that spring leaf unfolding delays by ~4 days for every degree increase in northern latitude (Hopkins, 1920), we observed a less pronounced response of only 1.98 days °N^−1^. Cheng et al. (2021) and Liu et al. (2019) reported decreases in the latitudinal variation of spring phenology over time, which was mainly attributed to spatial differences in the temperature sensitivity of spring phenology, with only slight advances under warming at low latitudes and strong advances at high latitudes. Consequently, these asymmetric changes in spring phenology across latitudes lower the spatial variation in budburst dates, which was also supported by our experimental results. We found a greater daylength limitation on budburst at low latitudes and daylength insensitivity at high latitudes, which would lead us to predict a larger advance in budburst dates at high latitudes relative to low latitudes under climate warming. Climate warming is thus likely to reduce spatial differences in budburst dates both across latitudinal and elevational gradients.

### Effects of Temperature and Daylength on Budburst

Consistent with previous studies, we observed significant advances in budburst with warming (Fu et al., 2013; Hänninen, 2016; Wu et al., 2022b). In addition to temperature, daylength has been suggested as another dominant factor regulating budburst dates (Basler and Körner, 2012; Way and Montgomery, 2015; Fu et al., 2019a). Since frosts may unexpectedly occur until late in spring, reliance on daylength might help plants to prevent precocious leaf-out and frost damage to young leaves (Zohner et al., 2020a). The forcing requirements of daylength sensitive species decrease with increasing daylength, thus delaying budburst under early warm spells (Fu et al., 2019a). On the other hand, warmer winters might also increase plants’ forcing requirements as a result of reduced chilling accumulation. Long daylength can compensate for insufficient chilling and promote budburst, allowing plants to use favorable spring conditions for photosynthesis (Basler and Körner, 2012; Vitasse, 2013; Way and Montgomery, 2015). Daylength can thus have a dual role, both delaying and advancing budburst under certain conditions, reducing the overall variation in budburst dates over time.

Recent study demonstrated the decreased daylength sensitivity of spring phenology along elevation (Wu et al., 2022b), which was consistent with our observations. Interestingly, we found a high daylength sensitivity at low latitudes and daylength-independent at high latitudes. This might partly be explained by the high chilling accumulation at high latitudes (Zhang et al., 2007; Wenden et al., 2020), whereby chilling compensates for daylength, leaving spring temperature (GDD) as the dominant factor regulating budburst dates. On the contrary, chilling accumulation was substantially reduced at low latitudes, causing daylength to play a more important role by interacting with temperature. The chilling threshold to break dormancy might vary across space as well, and trees from high latitudes might exhibit higher chilling requirements (Sawamura et al., 2017). Since warming rates increase with latitude (IPCC, 2021), chilling accumulation might also become insufficient at high latitudes in the future, and thus, daylength may affect future budburst dates at both high- and low-latitude regions. In line with experimental results, daylength effect was mainly found at low latitudes using *in situ* observations. In details, we found that variation in budburst dates that could be an indirect index of daylength effect on budburst as suggested by Zohner et al. (2016) was significantly reduced toward low latitudes that suggests a larger daylength limitation on budburst at low latitudes (Figure 6).

### Effects of Local Environment on the Phenological Responses to Climate Change

In our experiment, we found that twigs collected at high latitude and elevation sites showed earlier budburst than the low-latitude/low-elevation twigs when kept under the same temperature and daylength conditions, which was in agreement with Zohner et al. (2020b). As shown in Figure 5; Supplementary Figure S2, this might be explained by the longer chilling period that twigs from high latitude or elevation sites experienced before the collection, which might have led to a reduction in the heat requirement for budburst and a shorter time to budburst (Laube et al., 2013; Du et al., 2019). In addition, environment-induced adaptive plasticity might lead to lower heat requirements to achieve budburst in colder environments (Vitasse et al., 2010; Firmat et al., 2017).

Interestingly, although the timing to achieve budburst was shortened for the twigs collected in high latitude and elevation, the temperature sensitivity of budburst was significantly lower in twigs from high latitude and elevations. Similar results were reported using a remote sensing-based dataset (Gao et al., 2020). Plants growing in high latitudinal and altitudinal regions with higher temperature variance may have adapted to unstable temperature conditions by developing a growth strategy with a lower temperature sensitivity of budburst (Lechowicz, 1984; Wang et al., 2014). Another possible mechanism is the temperature threshold differences in phenological responses. In detail, plants from high latitudes and elevations may have adapted to lower temperatures and might be more responsive to low temperatures (here ~10°C) than plants from low latitudes and elevations. Thus, while plants from low latitudes and elevations need a lot of time to leaf-out at 10°C, plants from high latitudes and elevations might quickly respond to 10°C.

## Conclusion

The *in situ* phenological observations and the manipulative twig-cutting experiments demonstrate latitudinal patterns in spring leaf phenology and interactive effects of temperature and daylength on spring budburst of ginkgo twigs from different latitudes and elevations. Warming and longer daylength significantly advanced budburst, and the magnitude of the advancing trend was significantly different between latitudes and elevations. Interestingly, daylength only affected budburst at the low-latitude site, which might be caused by spatial differences in winter regime and local adaptive strategies of plants. This asynchronized response of budburst to climate change among latitudes and elevations—whereby low-latitude and low-elevation individuals are less responsive to climate change due to their inherent daylength sensitivity—implies that vegetation phenology might become more uniform across latitudes and elevations in the future. These shifts in spring phenological patterns along latitudinal/elevational gradients may have large effects on the structure and function of terrestrial ecosystems. Further studies on population-level differences in the interactive effects of temperature and daylength in a wide range of species are needed to improve our understanding of phenological changes under future climate warming.

## Data Availability Statement

The datasets that support the findings of the current study are available from the corresponding author on reasonable request.

## Author Contributions

ZW: conceptualization, methodology, software, formal analysis, resources, and writing—original draft preparation. YF: conceptualization, methodology, writing—reviewing and editing, supervision, project administration, and funding acquisition. Y-PZ: writing—reviewing and editing, supervision, and funding acquisition. SW, YG, and CL: data curation, visualization, and validation. JP and C-FL: investigation and writing—reviewing and editing. All authors contributed to the article and approved the submitted version.

## Funding

This study was supported by the National Funds for Distinguished Young Youths (grant no. 42025101), the International Cooperation and Exchanges NSFC-FWO (32111530083), the 111 Project (grant no. B18006), the General Program of National Nature Science Foundation of China (no. 31870190), and the Joint China-Sweden Mobility Program (grant no. CH2020-8656).

## Conflict of Interest

The authors declare that the research was conducted in the absence of any commercial or financial relationships that could be construed as a potential conflict of interest.

## Publisher’s Note

All claims expressed in this article are solely those of the authors and do not necessarily represent those of their affiliated organizations, or those of the publisher, the editors and the reviewers. Any product that may be evaluated in this article, or claim that may be made by its manufacturer, is not guaranteed or endorsed by the publisher.

## Footnotes

^1^ http://www.geodata.cn

^2^ http://westdc.westgis.ac.cn/data
