# Supplementary material for: Spatial Difference of Interactive Effect Between Temperature and Daylength on Ginkgo Budburst
Source: Front Plant Sci. 2022 May 10;13:887226. doi: 10.3389/fpls.2022.887226 (PMC9127872; doi:10.3389/fpls.2022.887226)
Supplement: Supplementary file 1 [file Data_Sheet_1.docx]

Supplementary Material

**Table. S1** Details of the selected phenological observation sites.

| ID | Sites | Latitude (°) | Longitude (°) | Duration (years) |
| --- | --- | --- | --- | --- |
| 1 | Baoding | 38.82 | 115.48 | 10 |
| 2 | Beijing | 39.99 | 116.27 | 17 |
| 3 | Hefei | 31.75 | 117.23 | 19 |
| 4 | Nanchang | 28.76 | 115.83 | 12 |
| 5 | Nanjing | 32.06 | 118.81 | 10 |
| 6 | Qinhuangdao | 39.83 | 119.53 | 12 |
| 7 | Shenyang | 41.83 | 123.57 | 12 |
| 8 | Xi'an | 34.21 | 109.03 | 22 |
| 9 | Yancheng | 33.26 | 119.92 | 10 |
| 10 | Zhenjiang | 32.21 | 119.44 | 16 |


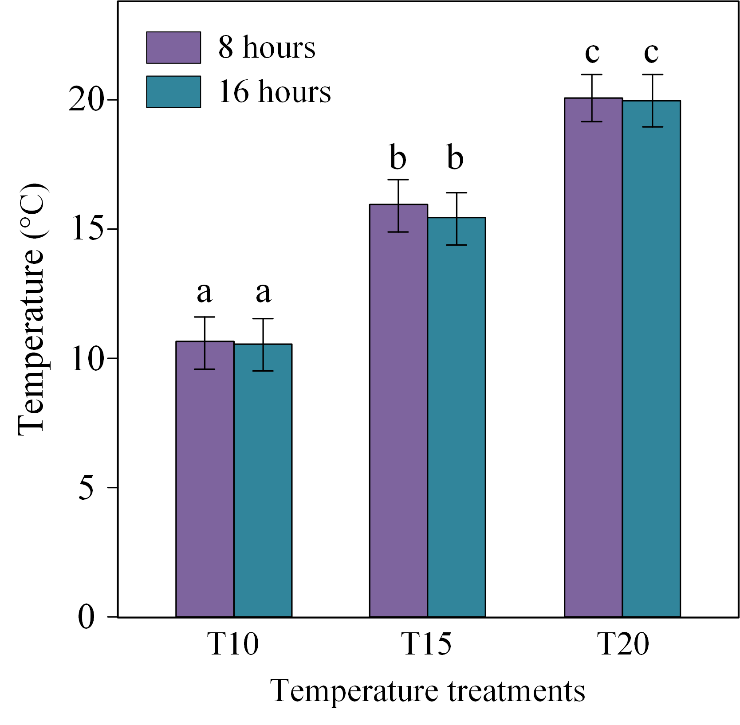


**Fig. S1** Air temperature recorded in each temperature and daylength treatment during the experiment (from 23 January to budburst date of the last twig). T10, T15 and T20 are the temperature treatments of 10, 15 and 20 °C. Error bars are standard errors. Same letters denote no significant difference (*P* > 0.05) in temperature between the daylength treatments.


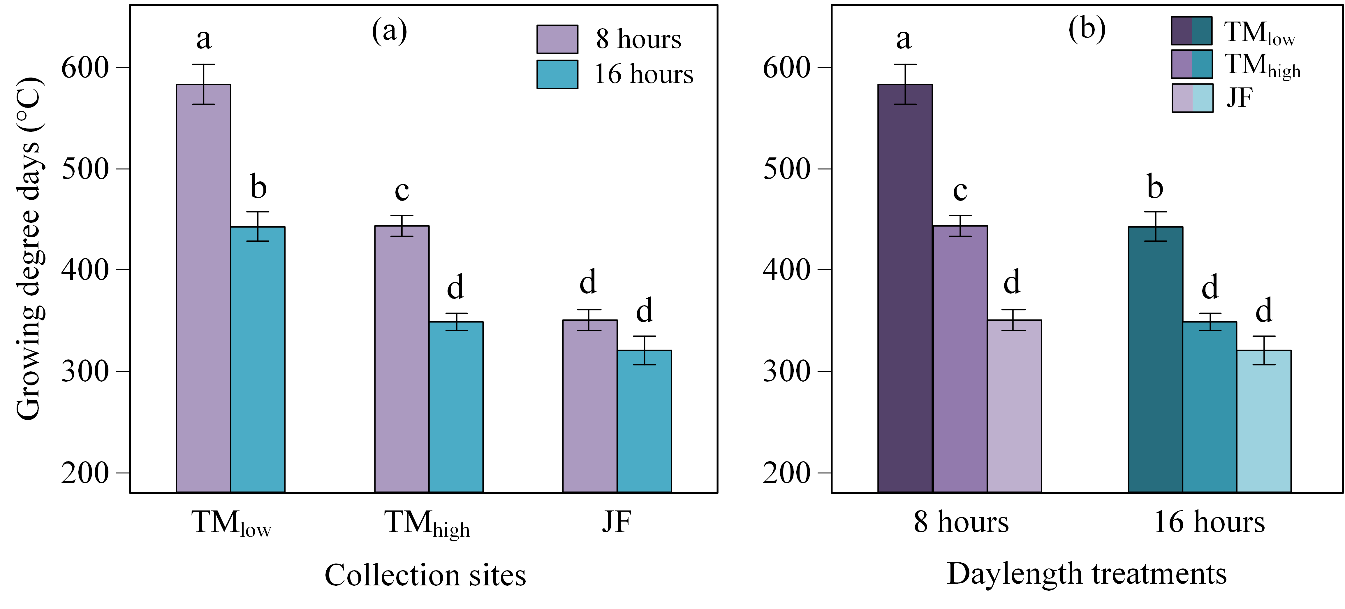


**Fig. S2** (a) Effects of daylength on growing degree days to achieve budburst in twigs from the three collection sites. (b) Same as panel a but grouped by daylength treatment. 8 and 16 hours refer to the daylength treatments in the climate chambers. TM_low_, TM_high_ and JF refer to the low-elevation and high-elevation collection site in Tianmu Mountain (low latitude), and the collection site in Jiufeng Mountain (high latitude), respectively. Different letters denote significant difference (*P* < 0.05) in growing degree days between the combinations of collection sites and daylength treatments.


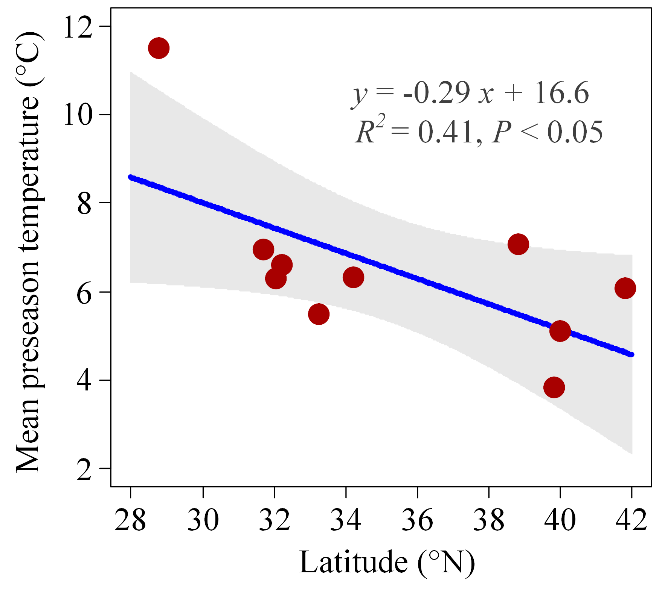


**Fig. S3** The relationship between mean preseason temperature and latitude.
